# Religious Beliefs, Trust In Public Figures, And Adherence to COVID-19 Health Guidelines among American Orthodox and Non-Orthodox Jews

**DOI:** 10.1007/s10943-022-01718-y

**Published:** 2022-12-14

**Authors:** Aaron D. Cherniak, Steven Pirutinsky, David H. Rosmarin

**Affiliations:** 1 Stockholm University, Stockholm, Sweden; 2 Touro College, New York, USA; 3 McLean Hospital/Harvard Medical School, Belmont, USA

**Keywords:** Pandemic, Spirituality, Medicine, Public health, Trust, Culture

## Abstract

The COVID-19 pandemic and resultant health crisis highlighted the lack of scholarly understanding of the effects of sociocultural factors and religious beliefs on compliance with public health guidelines. Orthodox Jews in particular were suspected of mistrusting medical experts and were singled out for alleged non-compliance with COVID-19 health guidelines. We surveyed American Jews (*N* = 1,141) during the early stages of the pandemic about their religious beliefs connected with the pandemic, trust in relevant public figures, and compliance with health guidelines to examine whether and how these factors are related. Generally, participants expressed high levels of trust in scientists, medical professionals, and religious leaders and a high degree of adherence to health guidelines. We examined how trust varies as a function of sociodemographic features, religious affiliation, and health-related religious beliefs (i.e., spiritual health locus of control). Overall, our research underscores the relevance of religious beliefs and trust in public figures to adherence to health guidelines and public health messaging.

## Introduction

Since December 2019, SARS-CoV-2 (COVID-19) has caused mass death and economic devastation across the globe (as of September 11, 2022, there have been 603,711,760 confirmed cases of COVID-19, including 6,484,136 deaths; World Health Organization, 2022). Numerous public health measures were implemented, at varying times and to differing degrees in different places, to minimize the spread of infection—e.g., campaigns and mandates about personal hygiene, social distancing, masking, building closures, lockdowns, and testing (Centers for Disease Control, 2022). Studies have shown that various sociocultural factors and beliefs may influence people’s adherence to health guidelines in the context of the crisis (Passavanti et al., 2021). Especially in times of uncertainty, sociocultural factors and core beliefs may affect how people seek out, accept, and act on information consistent with those beliefs (Mercier & Sperber, 2011; Van Bavel et al., 2020). It is crucial to understand the beliefs that underlie responses to health crises to enlist cooperation with health measures (Rosenfeld et al., 2021).

Data are mixed about how religious beliefs and trust in public figures relate to health behaviors (Shahin et al., 2019; Van Bavel et al., 2020), and studies show that these patterns may vary based on context (e.g., geographical, cultural, religious; Ogunleye et al., 2020). However, religious people and leaders have been accused of denying science and exacerbating the spread of COVID-19 (Stewart, 2020). In reality, these factors are connected in more complex ways. For example, a multinational study found that religious orthodoxy (using the 7-item Orthodoxy subscale of the Post-Critical Belief scale; Duriez & Hutsebaut, 2000) predicted lower adherence to COVID-19 health guidelines, though this was partially mediated by trust in science (Plohl & Musil, 2021). Public health campaigns have leveraged religious beliefs to promote health behaviors with relative success, and clergypeople are proven allies in these efforts, which explains why COVID-19 health messaging has featured religious and cultural factors prominently (Koenig, 2020; World Health Organization, 2020). We sought to examine religious beliefs and trust in public figures in relation to adherence to COVID-19 health guidelines among American Jews.

### American Jewry and COVID-19

The American Jewish population totals approximately 7–7.6 million, representing approximately 2% of the total US population (Dashefsky & Sheskin, 2020). The Jewish community is highly diverse in its religious practices, theological beliefs, and levels of societal engagement (Pew, 2021). Approximately 37% of American Jews identify as Reform, 17% as Conservative, 9% as Orthodox, and 36% do not identify with any particular denomination or identify with smaller groups (Pew, 2021). The highest concentration of American Jews lives in the New York-New Jersey area (Pew, 2021), where the pandemic hit the US hardest in its early stages (CDC, 2020). The Steinhardt Social Research Institute reported that between May 19 and July 30, 2020, 4% of American Jews had been infected with COVID-19, and approximately 15% had a “*close other* who *had been sick or had died* of COVID-19” (emphasis added; Saxe et al., 2021).

Orthodox and non-Orthodox Jews differ in ways that likely bear on their responses to health crises. For example, Orthodox Jews are less likely than non-Orthodox Jews to have received a college education (Pew, 2021), which constitutes a barrier to understanding and trusting health information and complying with public health measures (Friedman & Abensour, 2020). Moreover, Orthodox Jews are more likely to have greater numbers of children, have lower incomes, and live in denser housing (Pew, 2021) each of which increase the risk of disease transmission. In addition, Orthodox Jews report less use of technology (Hack, 2007), which was crucial to daily life during the pandemic (e.g., receiving updated health information, maintaining social contact despite restrictions on social gatherings; Ho et al., 2020). Orthodox Jewish communities are highly interdependent and collectivistic, and their communal life relies heavily on in-person gatherings (Heilman, 2000). Under ordinary circumstances, this communal environment provides complementary group resources (products of religious communal belonging), social identity resources (products of being accepted as a religious group member), and psychosocial resources (products of religious beliefs; Bankier-Karp & Shain, 2022; Hayward & Krause, 2014). However, measures adopted throughout the USA that prohibited or strictly limited public religious activities (Burke, 2020; VanderWeele, 2020), while deemed necessary to minimize the spread of COVID-19, had an outsized effect on Orthodox Jews, especially because of religious requirements for and reliance upon in-person prayer services that cannot be substituted by electronically mediated services (Bankier-Karp & Shain, 2022).

How have American Orthodox Jews fared during the COVID-19 pandemic? In some studies, Orthodox Jews reported particularly high levels of COVID-19-related stress and related impact on physical (Pirutinsky et al., 2021) and psychological health (Pirutinsky et al., 2020). Indeed, Orthodox Jewish communities, especially tight-knit Hasidic groups, experienced higher rates of COVID-19 mortality than other ethnic or religious groups, and numerous influential religious leaders died, which further negatively impacted communal morale (Stack, 2020). However, other studies show a conflicting pattern: Notwithstanding the decline in group resources, likely a result of the cessation of public religious life, Orthodox Jews actually reported high levels of resilience, including an increase in psychosocial aspects of religion relevant to coping (Aronson et al., 2022; Bankier-Karp & Shain, 2022; Graham et al., 2020).

While previous studies have reported that Jews demonstrated a high level of compliance with COVID-19 health guidelines (Pirutinsky et al., 2020), Orthodox Jews were explicitly singled out for their alleged non-compliance, including by politicians and major news outlets (Stack & Goldstein, 2020). Moreover, for long before the pandemic, Jews had been the long-time biggest target of anti-religion hate crime in the USA (Federal Bureau of Investigation, 2019). The COVID-19 pandemic, including the allegations mentioned above, brought a substantial increase in anti-Semitic incidents, including relating to conspiracy theories about the pandemic that echo old libels (Kantor Center, 2020). It is important to clarify rates of compliance among Orthodox Jews and to prevent an escalation in anti-Semitic sentiment.

### Spiritual Health Locus of Control

While studies have examined links between religion and health variables, research on the mechanism explaining the relationship between these variables is still developing. One important religious factor that may shape health behaviors is “spiritual health locus of control” – an extension of locus of control research generally (Hou et al., 2017; Rotter, 1966) that refers to the degree to which a person believes that God or a Higher Power is ultimately in control of one’s health (Gabbard et al., 1986). Findings about how these beliefs affect feelings of personal responsibility for one’s health, impact health behaviors, and influence coping behaviors have been mixed (for reviews, see Timmins & Martin, 2019; Oman, 2018), including differences between faiths and cultures (Shrauger & Silverman, 1971).

Some studies have linked spiritual health locus of control beliefs to positive outcomes, including healthy eating, physical activity, less smoking and alcohol consumption (Debnam et al., 2012), positive attitudes toward preventative medicine (Allen et al., 2014), better coping with end-of-life distress (Hexem et al., 2011), and greater collaboration in psychiatric treatment (Serfaty et al., 2020). Interestingly, Jackson and Coursey (1988) found that attribution of control over one’s health to God was correlated with an internal locus of control about health. This finding could indicate that believing in God’s providence over one’s health can actually increase the sense of religious obligation to adhere to health guidelines (Oman, 2018). Similarly, Shrauger and Silverman (1971) found that more frequent participation in religious activities predicted greater endorsement of an internal locus of control, which may suggest that more active members of a religion may have more “immature” or “sophisticated” faith that allows for integrated belief in God’s “support” *and* one’s own responsibility (Malony, 1988).

On the other hand, having a certain degree of faith in God’s control over one’s health may lead to an over-reliance on God to provide healing and passivity toward one’s health. Accordingly, several studies have demonstrated that “deferring control to God” may help reduce disease-related anxiety but ultimately lead to less healthy coping behaviors and poorer psychosocial outcomes (e.g., McLaughlin et al., 2013). It is important to understand this type of religious belief, given that they may underlie the degree to which individuals comply with health guidelines.

While spiritual health locus of control beliefs are highly relevant to responses to major crises, they have been understudied in the context of public health. Most of the literature on spiritual health locus of control deals with personal health decisions or behaviors (Oman, 2018). However, the extreme contagiousness of COVID-19 and the dramatic nature of the ensuing public health measures made this study, conducted during the pandemic, uniquely relevant, as it sought to identify the intersection between individuals’ beliefs and adherence to COVID-19 health guidelines.

### The Present Study

The present study examines religious beliefs, trust in public figures (scientists, medical experts, and religious leaders), and adherence to COVID-19 health guidelines in the American Jewish community. In particular, in light of the disparaging depictions of Orthodox Jews in the media—as allegedly, en masse, ignoring medical experts and flouting government regulations (Stack & Goldstein, 2020)—we sought to examine the actual levels of adherence and factors related to that adherence. In addition, we aimed to provide data relevant to the perceived differences between Orthodox and non-Orthodox Jews, especially surrounding issues of modernity and greater society, which can breed cross-denominational resentment (Sarna, 2019). Furthermore, we sought to bring data to bear to inform policy in faith communities. By identifying messengers trusted by American Jews and culturally relevant predictors of adherence to health guidelines, we hope to highlight the importance of ongoing trust-building and collaboration between public health officials and religious leaders, improve the dissemination of authoritative health information, and promote the effective implementation of public health measures.

We sought to identify whether trust in public figures and religious beliefs predicted adherence to COVID-19 health guidelines and whether these patterns differed between Orthodox and non-Orthodox Jews. Specifically, we examined trust in scientists, medical professionals, and religious leaders, spiritual health locus of control beliefs, and religious beliefs related to COVID-19. We expected greater trust in scientists and doctors to be associated with a higher degree of adherence to health guidelines and that trust in religious leaders would have no association.

There is a lack of understanding of the relevance of locus of control research to Jews due to a lack of research on this population (cf. Shrauger & Silverman, 1971). Consequently, we refrained from making overly specific hypotheses about spiritual health locus of control beliefs. Generally, there is reason to expect that Jews’ religious faith promotes health behaviors. Judaism emphasizes health and healing, including by obligating individuals to take joint responsibility with God to care for one’s health (Mishneh Torah, 4) and healing others, an ethical imperative that overrides even the most basic precepts of religious law (Rosner, 2003). Generally, we expected that Orthodox Jews, on account of their rootedness in Judaism’s rationalist-philosophic tradition, would view religious belief and trust in science as complementary. By contrast, non-Orthodox Jews may view religion as conflicting with science and rely only on the latter.

Trust in public figures and religious beliefs about health may have been particularly relevant during the early stages of the pandemic since COVID-19 was an unknown disease, a consensus among health experts was still taking form, and disinformation was (and still is) rampant (Capurro et al., 2021). Moreover, since no vaccine existed, compliance was particularly important.

## Method

### Procedure

Participants were recruited for a “Research Study on Jewish Community Responses to COVID-19” via Jewish organizations’ email lists, social media, and websites. Prospective participants were directed toward an anonymous online survey. After providing informed consent, they answered questions about demographics, COVID-19 risk factors, and religious beliefs. Inclusion criteria were: 18 years of age, self-identification as Jewish (any affiliation), proficiency in English (the language of the survey instruments), and access to a computer and the internet to facilitate participation in the study. Participants were informed that their participation was optional and would not be compensated. Ethical approval for the study was granted by the Institutional Review Board at (Institution of Author 2).

Data collection took place during the initial peak of the epidemic in the USA from March 29, 2020, until April 22, 2020. By March 29, there had been approximately 141,000 confirmed cases and 3,420 deaths in the USA, which rose to 840,500 confirmed cases and 47,400 deaths by April 22nd (Johns Hopkins University & Medicine, 2020). Worldwide, by March 29, there had been approximately 638,100 confirmed cases and 30,000 deaths; by April 22, there had been 2,476,000 confirmed cases and 169,100 deaths (World Health Organization, 2020).

### Participants

Demographic characteristics of our sample (*N* = 1,141) are summarized in Table 1. A significant minority of participants (*n* = 381, 21.4%) reported at least one risk factor for severe COVID-19, including being 65 years of age or older; having a history of cardiac disease, pulmonary disease, or immunosuppression; and residing in a long-term care facility. Regarding encounters with COVID-19, 47.2% had had contact with a confirmed or suspected case, 22.9% of participants had had a confirmed or suspected case themselves, and 56.1% knew a close other who had had a confirmed or suspected case.

**Table 1:** Sample characteristics

Total	*N* = 1,141
Age	Range: 18–92; *M* = 42.95, *SD* = 16.63
Gender	
Female	746 (65.4%)
Male	391 (34.3%)
Non-Binary	4 (.3%)
Religious Affiliation	
Orthodox Jews (e.g., Hasidic, Ultra-Orthodox, Modern Orthodox)	804 (70.5%)
Non-Orthodox Jews (e.g., Reform, Conservative, Reconstructionist)	337 (29.5%)
Median Household Income	$75,000–$100,000
Ethnic/Racial Identity	
White	94.6%
Other	5.4%
Family Status	
Married	68.6%
Single, never married	20.0%
Divorced	5.4%
Widowed	2.3%
Living with partner	2.1%

### Measures

#### Adherence to COVID-19 Health Guidelines

We presented participants with COVID-19 health guidelines from the Centers for Disease Control and Prevention (2020) and asked them to report their level of adherence using a Likert-type scale ranging from 1 (“not at all”) to 4 (“very much”).

#### Trust in Scientists, Medical Professionals, and Religious Leaders

We developed a series of items to assess participants’ trust in different public figures relevant to the pandemic—scientists, medical professionals, and religious leaders. Participants were asked to report the degree to which they agreed that these figures “act in the best interest of the public.” The items were rated on a 5-point Likert scale ranging from 1 (“disagree strongly”) to 5 (“agree strongly”).

#### Spiritual Health Locus of Control Scale

We included the Spiritual Health Locus of Control Scale (Holt et al., 2007) to assess beliefs about God’s control over one’s health. It uses a 5-point Likert scale ranging from 1 (“disagree strongly”) to 5 (“agree strongly”). Previous research has identified varying factor structures, including a two-factor structure (active and passive spiritual health locus of control; Holt et al., 2003) and a four-factor structure (spiritual life and faith, active spiritual, God’s grace, and passive spiritual; Holt et al., 2007), but the current study—e.g., understudied population of Jews, novel context of a major public health crisis—required an evaluation of a potentially different factor structure. A summary of our factor analysis is displayed in Table 2. We used a direct oblimin rotation and identified a three-factor structure that had better psychometric properties in our data than the other versions used previously (e.g., stronger factor loadings showing clearer factor structure). These components included (1) three items related to faith-based health beliefs (e.g., “Through my faith in God, I can stay healthy”), (2) five items related to collaborative health beliefs (e.g., “God and I share responsibility for my health”), and (3) two items related to submissive beliefs (e.g., “It’s ok not to seek medical attention because I feel that God will heal me”). Three items with insufficient loading or substantial cross-loading were excluded. We found excellent reliability for both the Faith-based subscale (*α* = 0.88 *ω* = 0.89) and the Collaborative subscale (*α* = 0.86, *ω* = 0.86), and moderate reliability for the 2-item Submissive subscale (*ρ* = 0.53; Eisinga et al., 2013).

**Table 2:** Factor Analysis of the Spiritual Health Locus of Control Scale in the Current Sample

	Item # in Holt et al., 2007	Factor loading			Reliability
Item		1—Faith	2—Collaborative	3—Submission	
If I lead a good spiritual life, I will stay healthy	2	**0.95**	− 0.10	-0.02	*α* = .88
Through my faith in God, I can stay healthy	1	**0.87**	0.03	-0.02	
If I stay healthy, it’s because I am doing what God wants	3	**0.86**	− 0.03	0.02	*α* = .86
God wants me to take care of my physical health	4	− 0.13	**0.89**	0.01	
God works through doctors to heal us	8	− 0.03	**0.85**	− 0.03	
Even though I trust God will take care of me, I still need to take care of myself	5	− 0.10	**0.84**	− 0.04	
God gives me the strength to take care of myself	6	0.14	**0.78**	0.00	
God and I share responsibility for my health	13	0.04	**0.65**	− 0.01	
There is no point in taking care of myself when it’s all up to God anyway	12	− 0.02	− 0.09	**0.83**	*ρ* = .53
It’s ok not to seek medical attention because I feel that God will heal me	11	− 0.02	0.05	**0.82**	

A three-factor solution was obtained using a direct oblimin rotation. Items were removed if they had no eigenvalues of > .4 on any factor or eigenvalues of > .4 on multiple factors. Three items were excluded according to this criteria

Bolded values indicate the factor to which each item belongs

#### Religious Beliefs about COVID-19

Participants were asked to respond to two separate statements: (1) “It is a religious duty to trust in God regarding COVID-19” and (2) “It is a religious duty to adhere to health guidelines regarding COVID-19” using a 5-point Likert scale ranging from 1 (“disagree strongly”) to 5 (“agree strongly”).

## Results

### Whom did Jews Trust about COVID-19?

#### Trust Reported by Overall Sample

Generally, participants expressed a substantial degree of trust in all sources (Table 3). While 75.3% of respondents agreed or strongly agreed that scientists act in the public's best interest, 11.8% disagreed or strongly disagreed. Similarly, 87.1% of participants agreed or strongly agreed that medical professionals act in the public's best interest, and 5.6% disagreed or strongly disagreed. Lastly, 74.3% of participants agreed or strongly agreed that religious leaders act in the public's best interest, and 10.2% disagreed or strongly disagreed. The overall sample reported greater trust in medical professionals (*M* = 4.36, *SD* = 0.87) than they did both in scientists (*M* = 3.99, *SD* = 1.02; *t*(1,273) = -17.468, *p* < 0.001) and in religious leaders (*M* = 3.93, *SD* = 1.04; *t*(1,269) = 12.076, *p* < 0.001), though a comparison of trust in scientists and religious leaders did not show a significant difference. Lastly, levels of trust in scientists, medical professionals, and religious leaders were all positively associated with one another (scientists-medical professionals: *r* = 0.15; scientists-religious leaders: *r* = 0.07; medical professionals-religious leaders: *r* = 0.12; *p* < 0.001 for all analyses).

**Table 3:** Adherence and Trust among American Jews

	Anchor	Overall	Orthodox Jews	Non-Orthodox Jews
Adherence to CDC guidelines	“Very much”	86.7%	86.4%	87.2%
	“Somewhat”	11.9%	13.0%	11.3%
	“Not very much”	0.8%	0.4%	0.9%
	“Not at all”	0.6%	0.2%	0.6%
Trust that ______ act in the best interest of the public				

Source	Anchor	Overall	Orthodox Jews	Non-Orthodox Jews
Scientists	“Strongly agree”	36.0%	30.1%	52.4%
	“Somewhat agree”	39.3%	40.4%	36.2%
	“Neither agree nor disagree”	12.9%	15.3%	6.3%
	“Somewhat disagree”	9.0%	10.9%	3.7%
	“Strongly disagree”	2.8%	3.3%	1.4%
Medical Professionals	“Strongly agree”	52.9%	49.3%	63.1%
	“Somewhat agree”	34.2%	35.6%	30.3%
	“Neither agree nor disagree”	7.4%	8.5%	4.1%
	“Somewhat disagree”	4.1%	4.9%	1.8%
	“Strongly disagree”	1.5%	1.7%	0.7%
Religious Leaders	“Strongly agree”	36.6%	42.7%	19.3%
	“Somewhat agree”	37.7%	34.3%	47.2%
	“Neither agree nor disagree”	15.5%	13.5%	21.2%
	“Somewhat disagree”	7.4%	6.7%	9.3%

#### Which Other Sociodemographic Variables Predicted Differences in Trust?

Next, we examined trust in relation to other sociodemographic variables. Men and women reported similar levels of trust in scientists and medical professionals, but men reported greater trust in religious leaders (*F*(2,1,023) = 5.586, *p* < 0.005). Older adults (age 65 + years) expressed more trust in scientists (*F*(1,1,029) = 10.659, *p* < 0.001), more trust in medical professionals (*F*(1,1,030, 5.974, *p* < 0.05), and less trust in religious leaders (*F*(1,1,024) = 16.246, *p* < 0.001) than non-seniors. Similarly, individuals reporting elevated risk to severe effects of COVID-19 expressed more trust in scientists (*F*(1,1,029) = 10.586, *p* < 0.001) and medical professionals (*F*(1,1,030) = 5.703, *p* < 0.05) but less trust in religious leaders (*F*(1,1,024) = 4.123, *p* < 0.05) than those who did not report risk factors.

#### Were there Inter-Denominational Differences in Trust?

When analyzing each denomination separately, a different pattern emerged. First, we examined whether there were significant differences in the trust that each group expressed in each figure, then we compared the two groups directly. Each group expressed the highest levels of trust in medical professionals, but the groups differed in the trust they expressed in scientists and religious leaders, which had been a null effect in the overall sample. Among Orthodox Jews, trust in medical professionals (*M* = 4.26, *SD* = 0.93) was greater than both trust in religious leaders (*M* = 4.07, *SD* = 1.08; *t*(755) = 4.108, *p* < 0.001) and trust in scientists (*M* = 3.83, *SD* = 1.04; *t*(757) = 14.674, *p* < 0.001), and, in turn, trust in religious leaders was greater than trust in scientists (*t*(754) = 4.650, *p* < 0.001). Among non-Orthodox Jews, trust in medical professionals (*M* = 4.35, *SD* = 0.73) was also greater than both trust in scientists (*M* = 4.34, *SD* = 0.87; *t*(270) = 5.267, *p* < 0.001) and trust in religious leaders (*M* = 3.71, *SD* = 0.98; *t*(268) = 12.064, *p* < 0.001). However, non-Orthodox Jews reported greater trust in scientists than in religious leaders (*t*(268) = 9.243, *p* < 0.001).

In direct comparisons between the groups, non-Orthodox Jews expressed more trust in scientists (*F*(1,1,029) = 49.654, *p* < 0.001) and medical professionals (*F*(1,1,030) = 19.165, *p* < 0.001) than did Orthodox Jews. These results held even when controlling for age, gender, and secular education levels for both scientists (*F*(1,804) = 29.55, *p* < 0.001) and professionals (*F*(1,804) = 7.44, *p* = 0.007). By contrast, Orthodox Jews reported greater trust in religious leaders (*F*(1,1,024) = 25.635, *p* < 0.001) even controlling for age, gender, and education (*F*(1, 800) = 11.34, *p* = 0.001). However, we underscore that both groups were generally trusting of all three sources of information (see Table 3).

### Did Jews Adhere to Health Guidelines?

#### Overall Sample

Levels of reported adherence to health guidelines were high—86.7% reported adhering “very much,” and an additional 11.9% reported adhering “somewhat” to health guidelines. Before comparing the adherence reported by Orthodox and non-Orthodox Jews, we examined other potentially relevant characteristics. There were no differences in self-reported adherence between men and women or between seniors and non-seniors. However, married individuals reported more adherence than unmarried individuals (*F*(1,1,120) = 5.201, *p* < 0.05) and individuals reporting elevated risk to severe effects of COVID-19 reported greater adherence to health guidelines (*F*(1,1,120) = 7.024, *p* < 0.005) than those without risk factors.

#### Were there Inter-Denominational Differences in Adherence?

Notwithstanding the differences in trust reported above, Orthodox and non-Orthodox Jews reported similar levels of adherence to COVID-19 health guidelines. Nearly 90% of each group reported adhering “very much” to CDC guidelines, and nearly all participants—99.4% of Orthodox Jews and 98.5% of non-Orthodox Jews—reported adhering at least “somewhat” to health guidelines. Rates did not significantly differ between Orthodox and non-Orthodox participants (X2(3, 1122) = 2.81, *p* = 0.42). A logistic regression predicting adherence also indicated that Orthodox and non-Orthodox did not significantly differ even when age, gender, and education was controlled for (*B* = − 0.38, *SE* = 0.23, *Wald X2*(1) = 2.64, *p* = 0.10).

### Do Trust and Religious Beliefs Predict Adherence to COVID-19 Health Guidelines?

We examined whether trust in public figures and religious beliefs about health predicted adherence in the overall sample and in each denomination. See Table 4.

**Table 4:** Associations between Trust, Adherence, and Health-Related Religious Beliefs

	Trust			Adherence
	Scientists	Medical professionals	Religious leaders	
Spiritual HLC				
Faith	− .14, *p* < .001	− .17, *p* < .001	.17, *p* < .001	-.07, p > .05
Collaborative	− .08, *p* < .01	− .14, *p* < .01	.22, *p* < .001	ns
Submission	− .16, *p* < .001	− .10, *p* < .005	ns	-.09, *p* < .01
Religious Duty				
To trust	− .22, *p* < .001	− .16, *p* < .001	.36, *p* < .001	ns
To adhere	.12, *p* < .001	.12, *p* < .001	.11, *p* < .001	.11, *p* < .001

#### Trust

For both Orthodox and non-Orthodox Jews, higher levels of trust in scientists and medical professionals were associated with greater adherence to COVID-19 health guidelines (OJ—scientists: *r* = 0.12, medical professionals: *r* = 0.13, both *p* < 0.001; non-OJ—scientists: *r* = 0.13, medical professionals: *r* = 0.14, both *p* < 0.001), whereas trust in religious leaders was not.

#### Spiritual Health Locus of Control

Next, we analyzed spiritual health locus of control beliefs, using the three-factor solution described in the methods section, and their link to adherence (see Table 2 for full list of items). Specifically, we examined: (1) faith-based beliefs—believing that health is attained through faith in God or wholesome spirituality; (2) collaborative beliefs—believing that responsibility for health is shared with God; and (3) submissive beliefs—believing that God alone will heal without medical intervention. In the overall sample, adherence was inversely related to faith-based beliefs (*r* = − 0.09, *p* = 0.01) and submissive beliefs (*r* = − 0.07, *p* = 0.05) but unrelated to collaborative beliefs. Next, we explored whether denominations differed in these patterns. Among non-Orthodox Jews, adherence was inversely related to faith-based beliefs, with a larger effect size than in the overall sample (*r* = − 0.19, *p* < 0.01). However, adherence was unrelated to submissive and collaborative beliefs. Among Orthodox Jews, adherence was inversely associated with submissive beliefs with a small effect size (*r* = -0.08, *p* < 0.05) and unrelated to faith-based and collaborative beliefs.

#### Beliefs about Religious Duties

Finally, we examined whether adherence was associated with religious beliefs related to COVID-19—whether one must trust in God regarding COVID-19 and whether one had a religious duty to adhere to medical guidelines. These views were positively correlated with each other (*r* = 0.28, *p* < 0.001), and there were no differences between Orthodox and non-Orthodox in this association. The belief in a duty to trust in God was unrelated to adherence, but the degree of belief in a religious duty to adhere to medical guidelines predicted greater adherence (*r* = 0.11, *p* < 0.001), with no significant difference in these associations based on religious affiliation.

## Discussion

Our study assessed links between religious factors and adherence to COVID-19 health guidelines in a large sample of American Jews. The critical finding is that there were high levels of trust in scientists, medical professionals, and religious leaders and, in the early stages of the pandemic, American Jews reported high levels of compliance with health guidelines. Despite minor intergroup differences in trust, there were no differences in adherence. Some, including in the media and politics, have singled out American Jews, a semi-visible minority, for alleged disregard for COVID-19-related measures (Stack & Goldstein, 2020). While American Jews have sociological characteristics and religious beliefs that may influence individual and communal responses to crises like COVID-19, our study indicates that overgeneralized accusations are unfounded.

We found links between trust in scientists, medical professionals, and clergy, which may imply a shared latent factor of trustfulness. Research often casts religion’s effects as monolithic (e.g., DeFranza et al., 2020), but we show that specific religious factors may have varied effects. In addition, despite intergroup differences in trust and religious observance, Orthodox and non-Orthodox Jews reported similar levels of adherence, pointing to alternate potential pathways to promoting health behaviors. For example, the association between trust in religious leaders and adherence may not have reached statistical significance, not because they are unrelated, but because of another variable that masks the association or ceiling effects in either variable. Our findings align with general differences between Orthodox Jews, whose identity may be based more on traditional beliefs, and non-Orthodox Jews, whose identity may relate more to culture and values (Pew Research Center, 2021).

Our study identifies specific religious beliefs that predict outcomes to a greater extent than religiosity generally. Furthermore, we found that the same belief can have both positive and negative effects depending on the context. While religious beliefs may promote better psychological coping, those same beliefs may lessen one’s concern about real dangers and inhibit behaviors necessary to protect oneself (Oman, 2018). For example, the view that life and one’s body are imbued with sacred qualities—“sanctification”—is associated with elevated self-rated health and positive body attitudes (Pargament & Mahoney, 2005). However, these same attitudes are related to taking fewer preventive health measures and experiencing greater subjective severity of physical symptoms (Benjamins et al., 2011; Krause et al., 2016). Notably, we found a positive association between the belief that one must trust in God regarding COVID-19 and the belief one has a religious duty to adhere to health guidelines. Apparently, believers do not experience a contradiction between trusting in God and acting to protect themselves. Future studies may assess whether religious beliefs predict not only self-reported health behaviors, but also actual health behaviors and epidemiological data (Nieuwsma et al., 2016).

Religiosity sits at the intersection of the multifaceted predictors examined in studies on beliefs about COVID-19 and adherence to health guidelines (e.g., Plohl & Musil, 2021). Many factors, including religious belief, affect how people navigate life events, perceived threats, and protective measures (Hood et al., 2018). Our findings indicate that religiosity and trust in clergy need not conflict with trust in sources of health information or adherence to health guidelines.

Studies of spiritual health beliefs have been mostly limited to Black protestants (Timmins & Martin, 2019). Our study highlights the culturally diverse expression of spiritual health beliefs, by examining an understudied population. Also, while prior studies have dealt with general health attitudes and screening measures in the context of preventative health education programs, this study is novel in its assessment of spiritual health beliefs related to a specific major health crisis—COVID-19. We suggest more research on health beliefs and behaviors in general, the role of religion, and particular health situations (e.g., personal health diagnosis, medical procedures, broader health threats).

Recognizing that trust and religious factors may affect public health measures and coping responses, many have advocated for implementing evidence-based strategies from social and behavioral science to improve responses to the crisis (Van Bavel et al., 2020). Our study provides preliminary descriptive data regarding American Jews’ response to COVID-19, including how they perceive public figures who convey health information. With the ongoing rollout of vaccination efforts, governments are increasing their involvement of community partners, such as clergy, to increase trust and reduce perceived tension between science and their faith (Miller, 2021). Future studies may use intervention-type or experimental designs to study how individuals adapt their religion-based health beliefs in response to new information from a trusted source. For example, people who feel that their religious belief conflicts with accepting information from scientists may be more receptive to trusted, local clergypeople who communicate that adherence to health measures may align with religious belief.

### Study Limitations

There were several limitations to this research. The study was cross-sectional, and it was conducted during the early stages of the pandemic. While we used a limited measure of adherence to health guidelines, we presented participants with up-to-date CDC guidelines before asking them to report their adherence. We asked participants whether it was a religious duty “to trust in God regarding COVID-19” leaving room understanding the question in different ways. Our question about trust asked only whether the source acted “in the best interest of the public,” but there are several other aspects of trust, such as credibility and honesty. Religion and politics are becoming intertwined to a greater degree in the USA (Hamid, 2021), and we did not assess political beliefs. At the time of the study, vaccines had not yet been developed; our findings cannot necessarily be extrapolated to predict the willingness to be vaccinated. We used a limited sampling method (some Orthodox Jews refrain or limit internet use), and we obtained a disproportionate number of women to men and a disproportionate number of Orthodox Jews than non-Orthodox Jews. Thus, our results are unlikely generalizable. However, the sample was novel and, similar to previous studies, we did not find gender differences in religious beliefs (e.g., Cherniak et al., 2021).

## Conclusion

Overall, our study examined American Orthodox and non-Orthodox Jews’ stance toward the COVID-19 pandemic, specifically relevant religious beliefs, trust in public figures, and adherence to public health guidelines. Despite its limitations, our study provides important preliminary evidence that American Jews expressed high levels of trust in scientists, medical professionals, and religious leaders and a high degree of adherence to health guidelines. Overall, our research underscores the relevance of religious beliefs and trust in public figures to adherence to health guidelines and public health messaging. Future studies can use our findings to continue probing the implications of trust and religious beliefs on adherence to health guidelines, and the relevance of these topics for improving religious communities’ responses to health crises.

## Data Availability

Data are available upon request.

## Code Availability

SPSS 26 was used.
